# Application of genus Cassia in the treatment of Constipation: A systematic review

**DOI:** 10.12688/f1000research.17893.1

**Published:** 2019-03-05

**Authors:** Muhammad Shahzad Aslam

**Affiliations:** 1 Department of Chemistry, Xiamen University Malaysia, Sepang, Selangor, 43900, Malaysia

**Keywords:** Cassia, Cassia fistula, Constipation, Pediatric gastroenterology

## Abstract

**Background**: Constipation is a common problem for children. There are a number of alternative treatments for constipation. Medicinal plants have a history of treating various ailments. *Cassia augustifolia* and *Cassia fistula* were commonly used species in the treatment of constipation. The aim of this study was to identify the role of genus *Cassia* in the treatment of Constipation

**Methods**: Articles include in the studies were randomized, clinical trials on pediatric functional constipation, of patients aged between 2–15 years, that were full-length open access articles. Meta-analysis was conducted using the Review Manager (RevMan) 5.3 software.

**Results**: *Cassia fistula* was partially effected in treating constipation however there is a need for improvement in the protocol of studies to reduce biases. These results were only limited to one species so it cannot be generalized among all species of Cassia.

**Conclusion**: *Cassia fistula* is partially effective in reducing the pain and consistency of stool during constipation among children.

## Introduction

Constipation is a clinical disorder attributed to ineffectual colonic impulsion and/or increased resistance to the proliferation of colonic matters ^1^. Approximately 20% of the world population suffers from chronic constipation ^2^. It is one of the most common pediatric problems ^3^. It was found to be the second most stated disorder in the field of pediatric gastroenterology. Treatment costs for children with constipation will be around three times higher than children without constipation in the United States ^4^. African American children, particularly girls, are greatly affected by constipation, which has been associated with poor hygiene conditions ^5^.

Commonly, constipation is treated by Cisapride in children ^6^, other treatments include polyethylene glycol 3350 and lactulose, however polyethylene glycol 3350 has been found to be more effective ^7^. Supplemented and non-supplemented yogurt helps in reducing abdominal pain and to enhance defecation frequency ^8^. It has been observed that different species of *Cassia* act an effective as a laxative such as *Cassia fistula, Cassia alata, and Cassia augustifolia* ^9– 11^. The genus *Cassia* is well known in alternative medicine as hepatoprotective ^12^, laxative, and in the treatment of ringworm infection ^13^, skin diseases ^14^ and leprosy ^15^. It has many pharmacological properties including acting as a hypolipidemic agent ^16^, anti-microbial ^17^, anti-fungal ^18^, and anti-cancer agent ^19^. Genus *Cassia* contains a number of bioactive compounds such as anthraquinone ^20^, tannin ^21^, coumarins ^22^, triterpene, volatile oil ^23^, phenolic glycoside ^24^, flavonoids ^25^ from different parts of the plant. Different species of *Cassia* possess laxative properties due to various anthraquinone derivative such as aloe-emodin, rhein, chrysophanol and chrysoobtusin. In this review, we systematically assessed the laxative potential of different species of Cassia.

## Methods

### Literature search strategy

**Figure 1.**
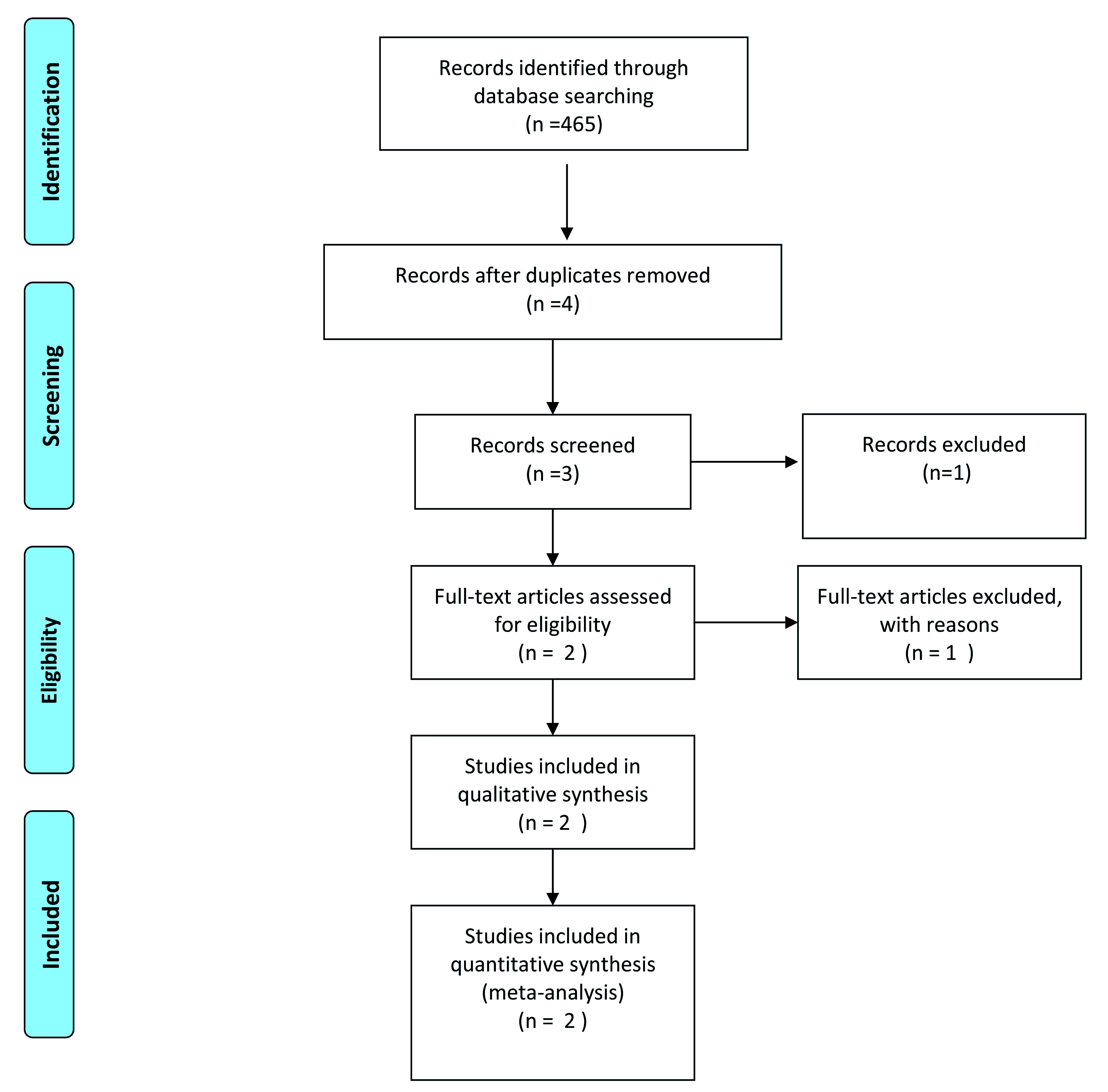
PRISMA flow diagram.

A systematic literature search was conducted in accordance with the Preferred Reporting Items for Systematic Reviews and Meta-Analyses (PRISMA) guidelines. Using the keywords (Senna AND Laxatives AND Clinical trial) (Cassia AND Laxatives AND Clinical trial) (Cassia AND Clinical trial) (Senna AND Clinical trial) and publication range from 01 January 1960 till 31 December 2018 for identification of the records. Table 1 shows the search strategy for PubMed Central. During screening of the records only full-length open access articles were considered. Abstract only or closed access articles were excluded ^11^. Only articles involving children aged between 2–15 were included. All review articles, *in-vivo* studies and those >10 years from the search data were excluded. A preliminary search of the PubMed, CNKI, Scopus, Web of Science, Google Scholar and PsycINFO databases and digital archive such as PubMed Central, yielded 2207 papers published in English from the last ten years. Duplicate and irrelevant articles were removed (n=2203). One article was further removed during screening due to closed access (n=3). One publication was removed because the article did not meet the eligibility criteria (n=2). A PRISMA Flow Diagram is given in Figure 1.

**Table 1.** Search strategy.

PubMed Central	
(“cassia”[MeSH Terms] OR “cassia”[All Fields]) AND (“clinical trial”[All Fields] OR “clinical trials as topic”[MeSH Terms] OR “clinical trial”[All Fields]) AND (“1960/01/01”[PubDate]: “2018/12/31”[PubDate])	((“cassia”[MeSH Terms] OR “cassia”[All Fields]) AND (“laxatives”[All Fields] OR “laxatives”[MeSH Terms] OR “laxatives”[All Fields])) AND (“clinical trial”[All Fields] OR “clinical trials as topic”[MeSH Terms] OR “clinical trial”[All Fields]) AND (“1960/01/01”[PubDate] : “2018/12/31”[PubDate])
(“Senna”[MeSH Terms] OR “Senna”[All Fields]) AND (“clinical trial”[All Fields] OR “clinical trials as topic”[MeSH Terms] OR “clinical trial”[All Fields]) AND (“1960/01/01”[PubDate]: “2018/12/31”[PubDate])	((“senna plant”[MeSH Terms] OR (“senna”[All Fields] AND “plant”[All Fields]) OR “senna plant”[All Fields] OR “senna”[All Fields]) AND (“laxatives”[All Fields] OR “laxatives”[MeSH Terms] OR “laxatives”[All Fields])) AND (“clinical trial”[All Fields] OR “clinical trials as topic”[MeSH Terms] OR “clinical trial”[All Fields]) AND (“1960/01/01”[PubDate] : “2018/12/31”[PubDate])

### Literature screening

Identification of articles were performed at level 1 using the search strategy as mentioned in Table 1. Duplicate articles, irrelevant articles such as polyherbal formulation, review articles, or any article other than Cassia or Senna were removed at level 2. Only four ^4^ articles were identified as being relevant. One record was excluded due to not being a full text article. Abstracts were being reviewed for the following inclusion and exclusion criteria at level 4 and one article was removed for not meeting the eligibility criteria i.e. Randomized, clinical trial on Constipation, full-length open access articles, Pediatric Functional Constipation (age range: 2–15 years).

### Eligibility criteria

***Types of studies.*** The author has selected studies of randomized open label, prospective, controlled, parallel-group clinical trial for meta-analysis. Baseline characteristics of randomized trials of studies included on pediatric functional constipation are presented in Table 4. Characteristics of the studies included are mentioned in Table 5.

***Types of participants.*** The author included studies involving patients (aged 2–15 years) with Functional constipation. The diagnosis of Functional constipation was according to according to the Rome III criteria ^26^. Inclusion and exclusion criteria were based on Study design, participants, intervention, outcome (SPIO) criteria and indicated in Table 2.

**Table 2.** Study design; participants; intervention; outcome (SPIO) criteria.

	Inclusion Criteria	Exclusion Criteria
**Study design**	Randomized, clinical trial on Constipation, full-length open access articles, Pediatric Functional Constipation	All review articles, irrelevant articles, Exclude abstract only articles, clinical trial on adults.
**Participants**	Children (age range: 2–15 years)	Adults
**Intervention**	*Cassia fistula* was delivered to Pediatric with Functional Constipation	
**Outcomes**	Role of the *Cassia fistula* emulsion in Pediatric Functional Constipation	

***Types of interventions.*** Included studies were focused on the role Cassia in the treatment of Functional constipation. Unfortunately, there were only two studies identified. 

***Types of outcomes.*** Eligible studies included consisted of the following outcomes: improvement in the episodes of fecal incontinence per week, improvement in the episodes of retentive posturing per week, improvement in the average of severity of pain of defecation (by VAS), improvement in defecation frequency per week, patient’s drug compliance and improvement in the average of consistency of stool defecated (by VAS).

### Methodological quality assessment (MQ)

**Figure 2.**
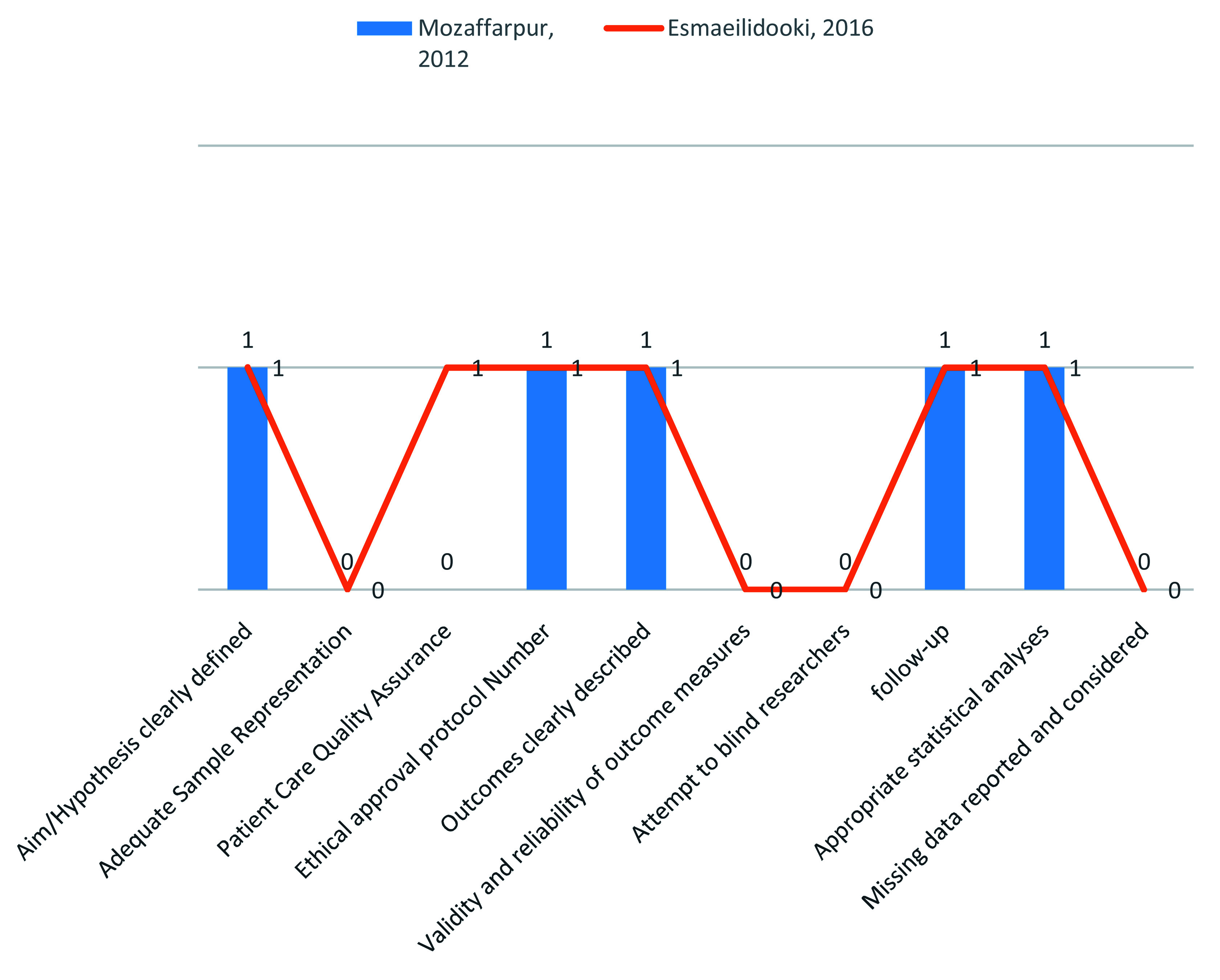
Methodological quality assessment of the 2 studies included in the meta-analysis (0=No/not reported, 1=Yes).

Methodological quality assessment was made on the basis of following criteria. 1) Aims and Hypothesis clearly defined, adequate sample representation, patient care quality, ethical approval protocol, outcomes assessment, validity and reliability of outcome measure, attempt to blind researcher, follow-up, appropriate statistical analysis and missing data reported. Ten item defined evaluation of methodological quality (MQ) is presented in Figure 2. Risk of Bias were assessed using Cochrane collaboration's tool on the basis of the following criteria such as selection bias, performance bias, attrition bias, reporting bias and miscellaneous. Cochrane Collaboration’s tool for assessing the risk of bias was used and the results are presented in Table 6 ^27^.

### Data extraction

The following data were extracted according to study characteristics (e.g., first author, year of publication, search dates, and number of included studies), patient characteristics (functional constipated children, aged between 2–15), sample size, study type (e.g., Randomized open label, prospective, controlled, parallel-group clinical trial study), randomization methods (e.g., "systematic randomization and simple randomization”) and outcome measures/variables (e.g., improvement in defecation frequency per week, improvement in the episodes of fecal incontinence per week, improvement in the episodes of retentive posturing per week, improvement in the average of severity of pain of defecation (by VAS), improvement in the average of consistency of stool defecated (by VAS) and patient’s drug compliance). Data extraction was performed by Muhammad Shazad Aslam. Transcripts were analysed, coded and data was extracted using the demo version of qualitative data analysis software Atlas.ti 8.0 . Table 3 represent all the data that was extracted. All the meta-data are available as Dataset 1.

**Table 3.** Data extraction.

Characteristics	Seyyed Ali Mozaffarpur	Mohammad Reza Esmaeilidooki
Publication Year	2012	2016
Type of Study	Randomized, clinical trial	This randomized open label, prospective, controlled, parallel- group clinical trial study
Age	Age between 4–13 years	Aged between 2 – 15 years
Randomization	systematic randomization	Simple randomization
Total Sample Size	81	51
Variables	Frequency of defecation, consistency of stools, and severity of pain during defecation, retentive posturing and fecal incontinence per week	Improvement in defecation frequency per week, improvement in the episodes of fecal incontinence per week, improvement in the episodes of retentive posturing per week, improvement in the average of severity of pain of defecation (by VAS), improvement in the average of consistency of stool defecated (by VAS) and patient’s drug compliance
Length of each contact with the participant/caregivers	Clinical efficacy and tolerance were assessed using weekly sheets, parents completed every night. They were given three sheets (included seven questions in seven columns) to complete them daily for 3 weeks.	During the study, we had regular phone calls with the parents to check the probable complications, treatment (taking the prescribed drugs) and data ﬁlling process. If there were any serious questions or problems, we visited the child. At the end of 4 weeks of treatment, the children were visited and the filled out forms were taken and evaluated.
Blinding of Experiment	The investigators, the children and their parents were aware of the study group assignment.	Fortunately, due to the developing socioeconomic conditions of the people in these regions in recent years, we were able to keep in touch with all the patients during the study period by phone call.
Diagnosis	Rome III criteria of functional constipation	Rome III criteria of functional constipation
Diagnostic test	Paraclinics like anorectal manometry, thyroid function tests, anti-tTG, and other laboratory tests	thyroid function tests, anti-tTG, and etc. If it remained any doubt, barium study and anorectal manometry would be performed

**Table 4.** Baseline characteristics of randomized trials of studies included in pediatric functional constipation.

Variable in Treatment (T)	Mozaffarpur, 2012 (T)	Esmaeilidooki, 2016 (T)	Mozaffarpur, 2012 (C)	Esmaeilidooki, 2016 (C)	Variable in Control (C)
**Sample size in Treatment (N)**	41	52	40	57	**Sample size in Control (N)**
**Age, months, Mean(±SD)**	69.4(±24.3)	64.6(±25.2)	65.9(±19.1)	55.2(±31.2)	**Age, months, Mean(±SD)**
**Sex, Male, (n) (%)**	29(70.7%)	33	23(57.5%)	30	**Sex, Male, (n) (%)**
**Weight, Kg Mean(±SD)**	21.7 (±7.2)	20.5(±7.2)	20.7(±7.8)	18.5(±8.9)	**Weight, Kg(±SD)**
**Duration of constipation,** **months, Mean(±SD)**	34.2(±25.9)	31.1(±24.6)	30.8(±22.8)	23.5(±21.8)	**Duration of constipation,** **months, Mean(±SD)**
**Defecation ≤ 2 per week, n (%)**	32(78%)	41	30(75%)	52	**Defecation ≤ 2 per week, n (%)**
**Incontinence, n (%)**	31(75.6%)	34	27(67.5%)	37	**Incontinence, n (%)**
**History of previous treatment,** **n (%)**	32 (78%)	43	28(70%)	51	**History of previous treatment,** **n (%)**
**Retentive posturing, n (%)**	32(78%)	40	29(72.5%)	37	**Retentive posturing, n (%)**

**Table 5.** Study characteristics.

Author, year	Study Design	Hypothesis	Statistical analysis	Software	Outcomes
**Mozaffarpur, 2012**	Randomized, clinical trial	The author hypothesized that *Cassia fistula* emulsion (CFE) would be as effective or better than Mineral oil (MO) in treating FC.	The statistical analyses included the determination of means and Standard deviation (SDs), t test, χ2 test, ANOVA repeated measures and Fisher’s exact test, with significance accepted at the 5% level.	SPSS (version 17),	CFE was most effective than MO in the 3-week treatment of children with FC.
**Esmaeilidooki, 2016**	Randomized, clinical trial	N/A	The statistical analyses included the determination of means and SDs, t test, χ2 test, ANOVA repeated measures and Fisher’s exact test, with significance accepted at the 5% level.	SPSS IBM20 and STATA 11.2	Significant improvement when compared with the control group however unable to find substantial evidence of the role of identified bioactive compounds due to limitation as it requires further investigation

**Table 6.** Cochrane Collaboration’s tool for assessing risk of bias.

Study (author, year)	Selection bias	Performance bias	Detection bias	Attrition bias	Reporting bias	Other bias
**Mozaffarpur, 2012**	Unclear	Yes	Yes	No	No	Unclear
**Esmaeilidooki, 2016**	Unclear	Yes	Yes	No	No	Unclear

### Statistical analysis

Meta-analysis was conducted using the Review Manager (RevMan) 5.3 software ^28^. The summary measures were reported as odds ratios (ORs) or as a standard mean difference (SMD) with 95% confidence intervals (CI). The presence of heterogeneity among trials was assessed using the Chi-square test, and the extent of the inconsistency was measured by I2 statistics. Output file from RevMan is available as Underlying data ^29^.

## Results and discussion

**Figure 3.**
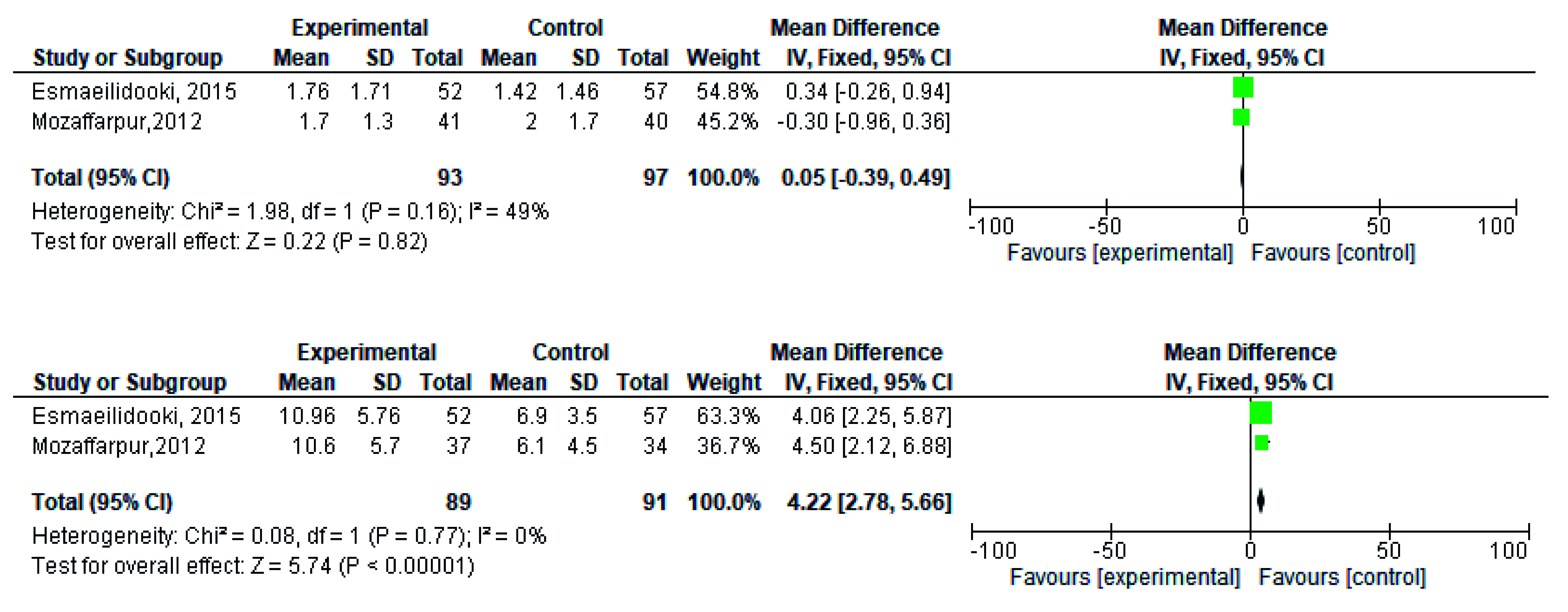
Forest plot in defaecation before and after treatment.

**Figure 4.**
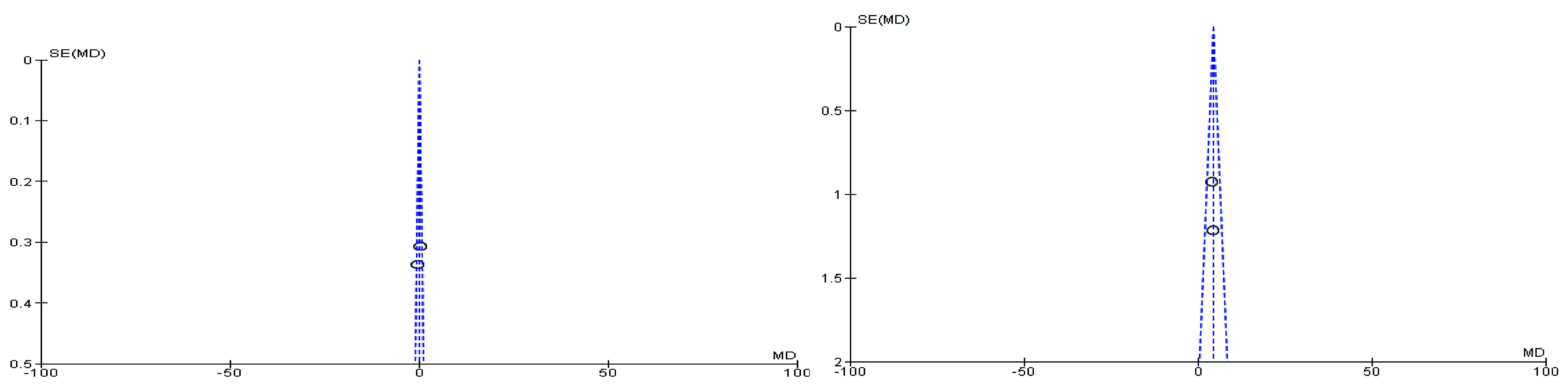
Funnel plot showing overall standardized mean difference in defaecation before and after treatment.

**Figure 5.**
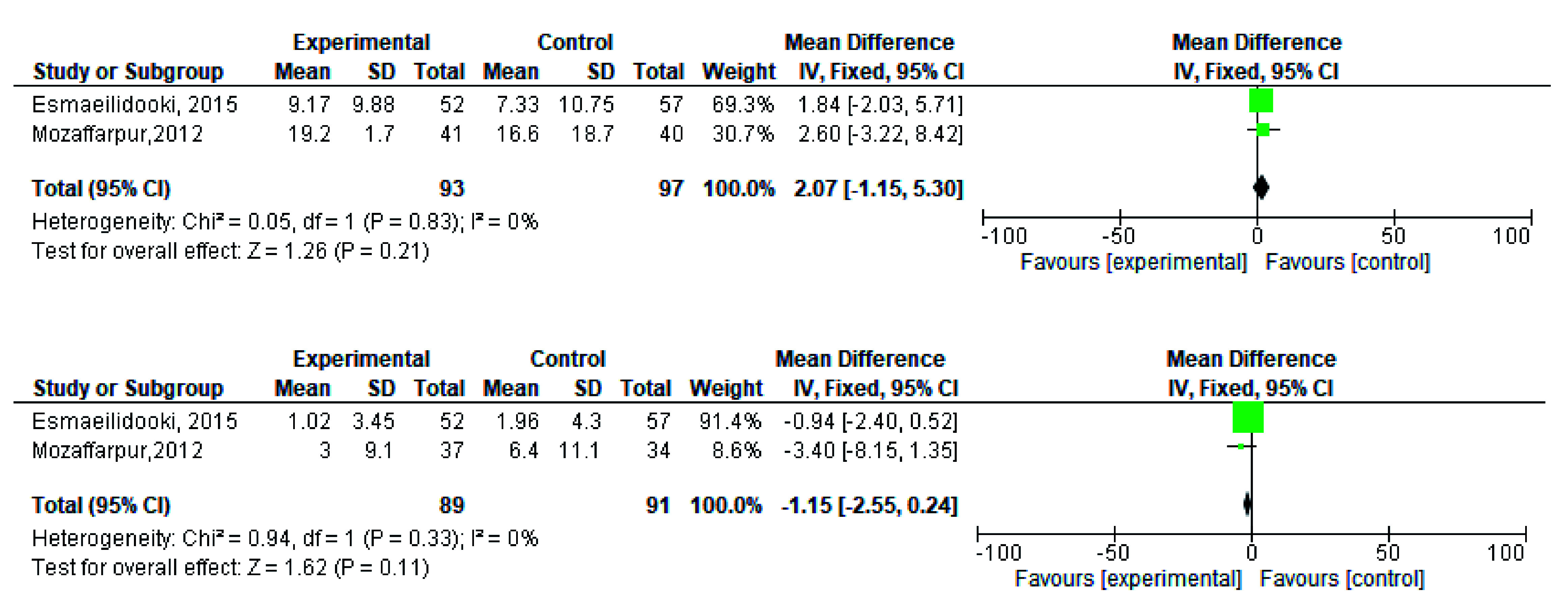
Forest plot in fecal incontinence before and after treatment.

**Figure 6.**
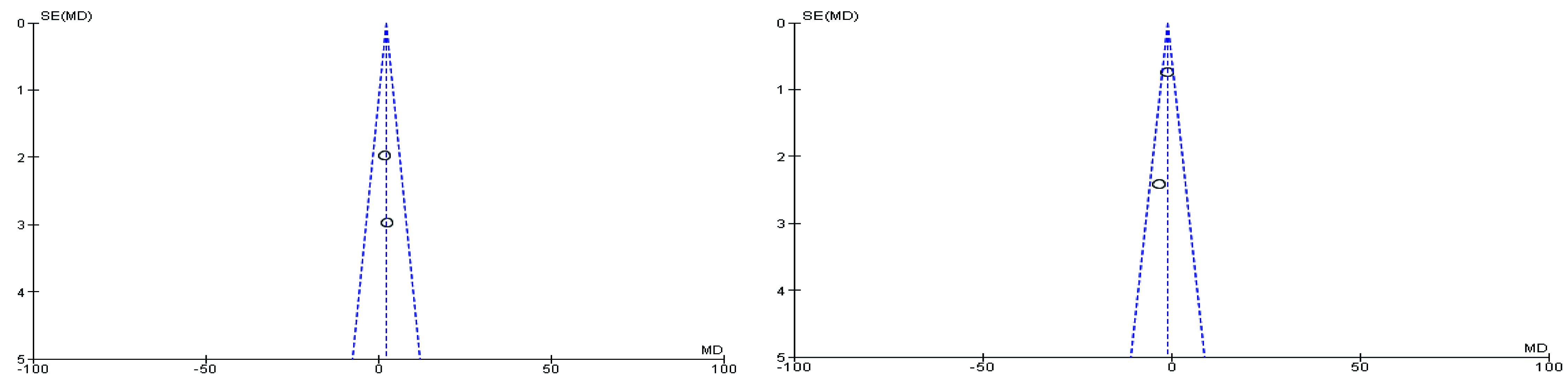
Funnel plot showing overall standardized mean difference in fecal incontinence before and after treatment.

**Figure 7.**
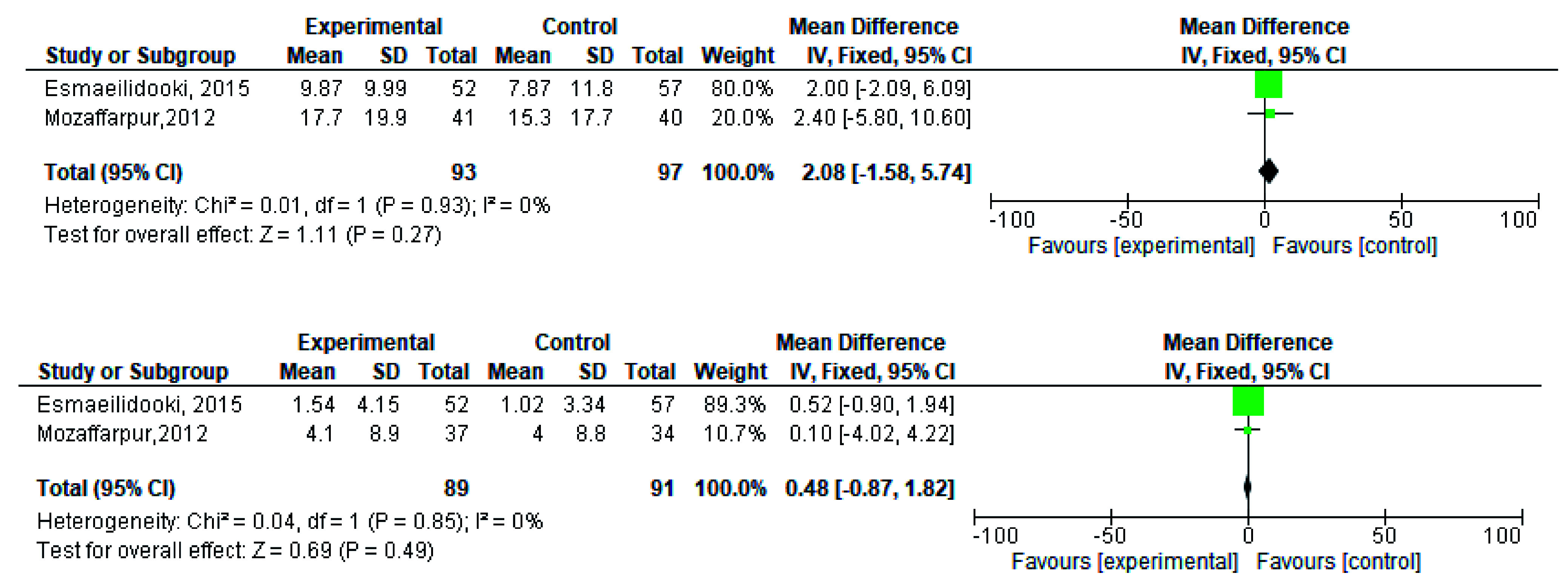
Forest plot in retentive posturing before and after treatment.

**Figure 8.**
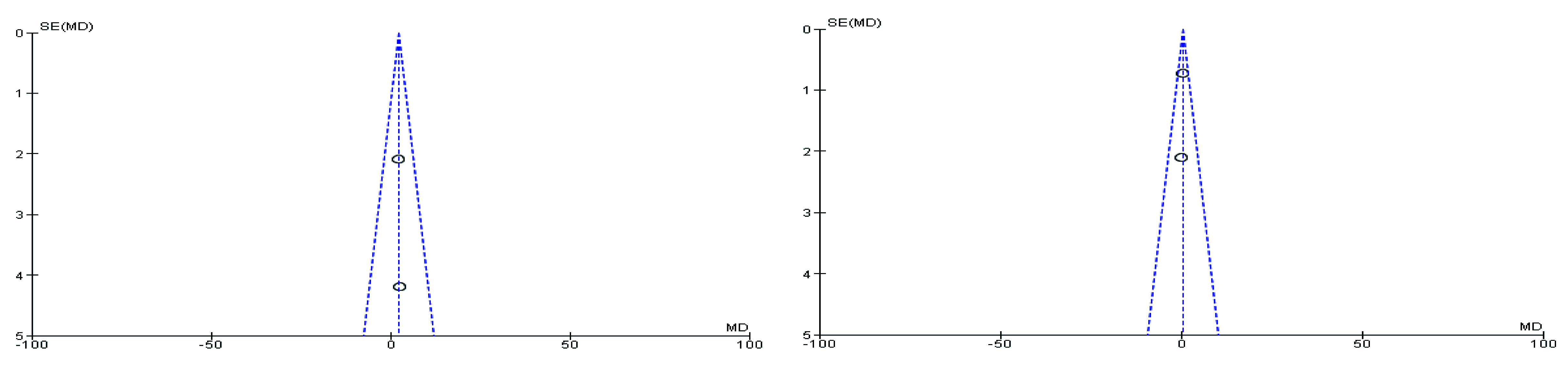
Funnel plot showing overall standardized mean difference in retentive posturing before and after treatment.

**Figure 9.**
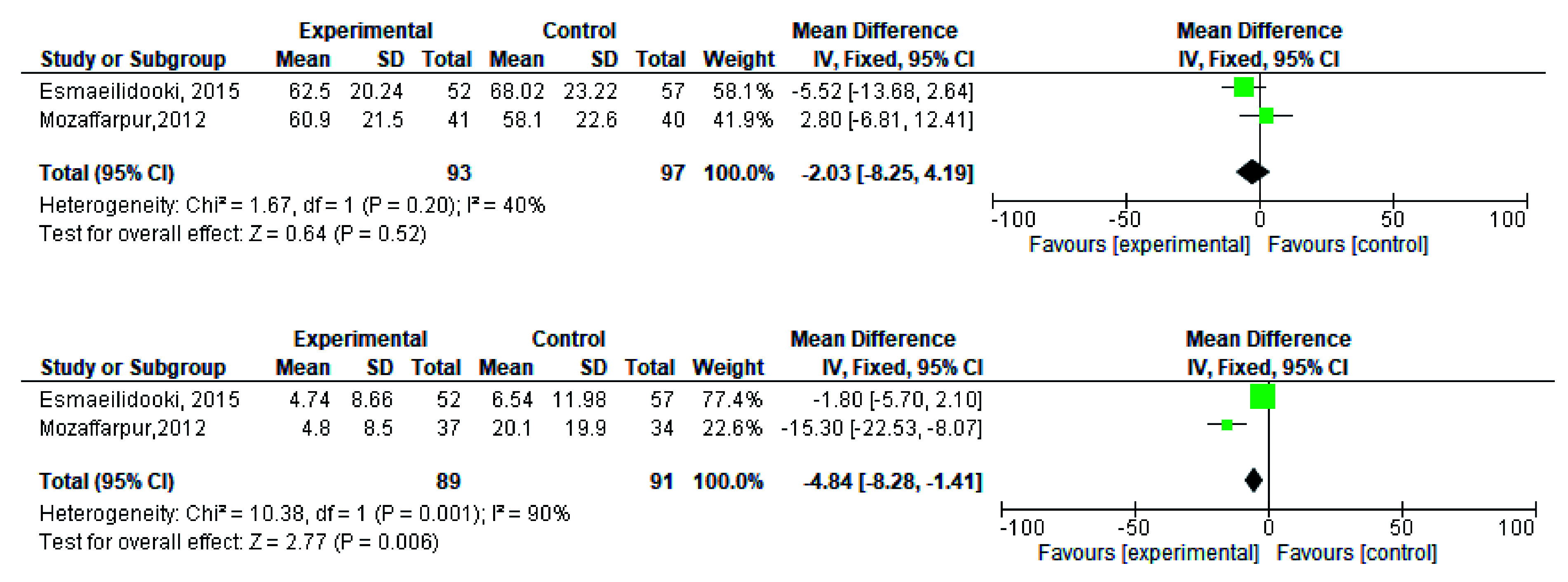
Forest plot in severity of pain before and after treatment.

**Figure 10.**
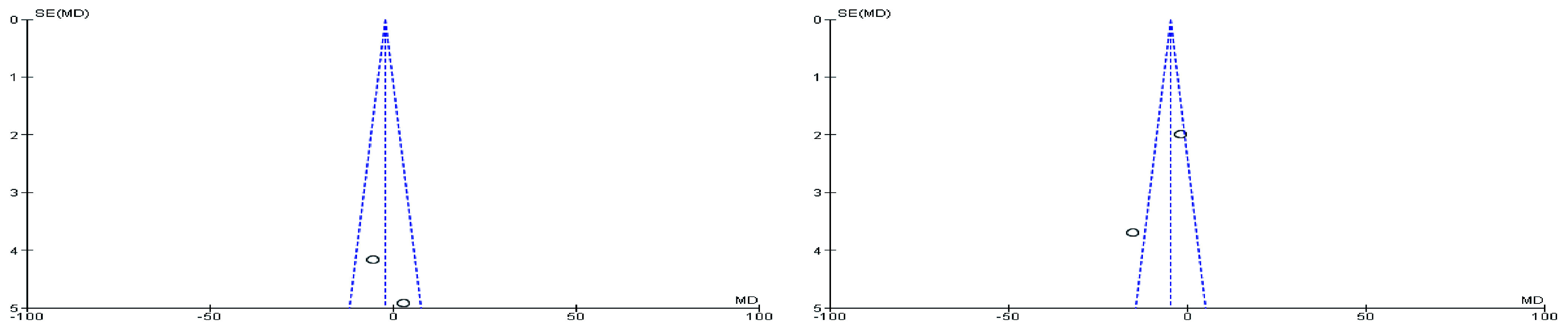
Funnel plot showing overall standardized mean difference in severity of pain before and after treatment.

**Figure 11.**
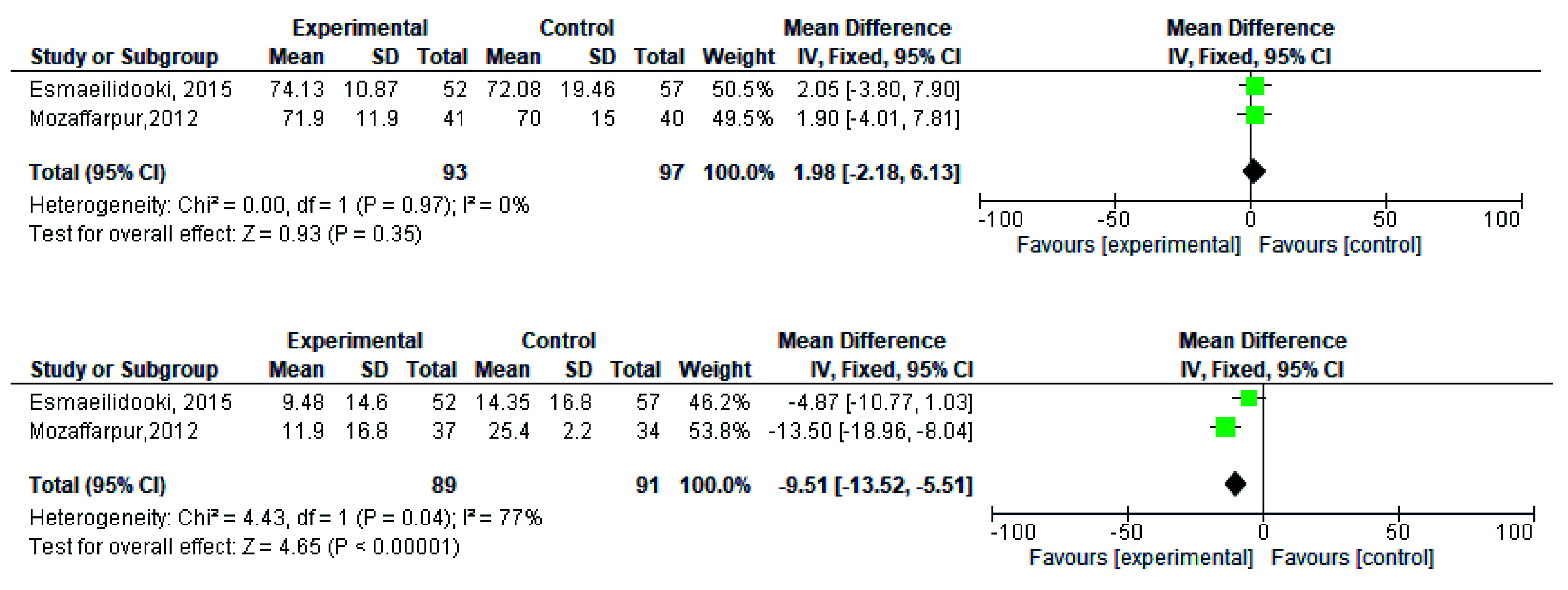
Forest plot in consistency of stool before and after treatment.

**Figure 12.**
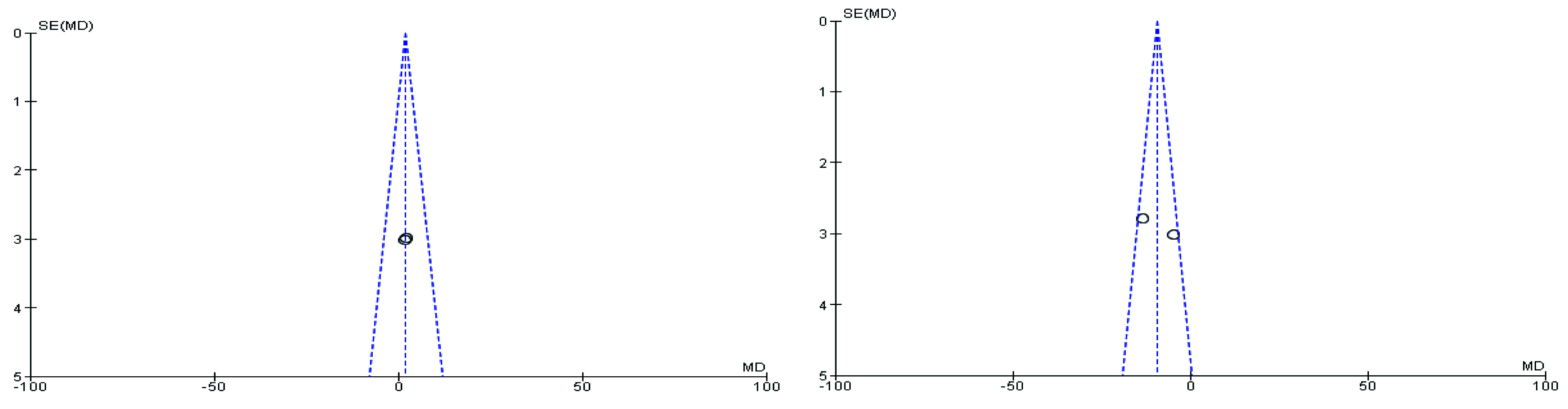
Funnel plot showing overall standardized mean difference in consistency of stool before and after treatment.

**Figure 13.**
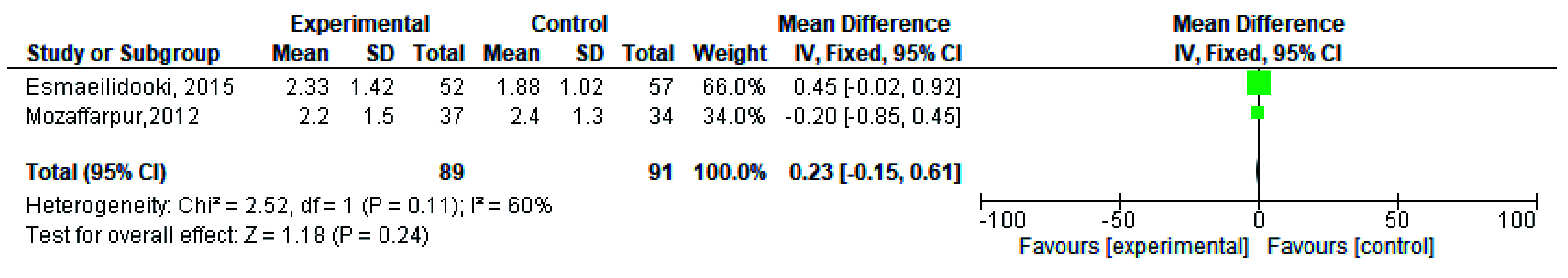
Forest plot in acceptance and tolerance after treatment.

**Figure 14.**
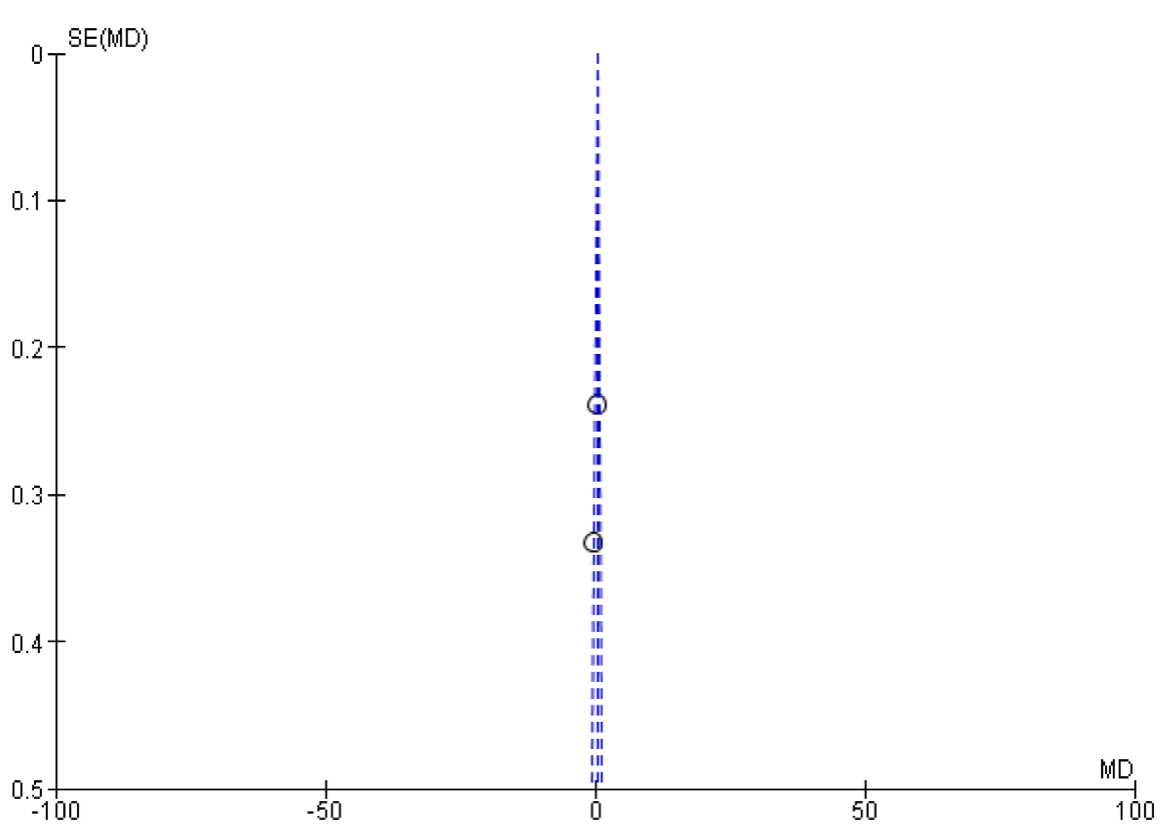
Funnel plot showing overall standardized mean difference in acceptance and tolerance after treatment.

Both of the selected studies were not blinded during intervention and outcome assessment that will result in performance bias and detection bias respectively. These biases occur where the investigators know about the participant's treatment group. Performance bias can also refer to the fact that participants can change their responses or behaviour if they know which group they are allocated in. Blinding of outcome assessment may decrease the risk of the investigator or participant being aware of the treatment that a patient is receiving. If the participants and the caregivers are aware of the intervention and outcome that may affect the behavior of the participants, these behavioral changes may affect the performance of the treatment. Clinical trials on adults were also excluded, such as a randomized clinical trial of a phytotherapeutic compound containing *Pimpinella anisum, Foeniculum vulgare, Sambucus nigra*, and *Cassia augustifolia* for chronic constipation ^9^. Results of both included studies were non-significant when comparing their baseline characteristics of pediatric functional constipation as presented in Table 5. During analysis of study characteristics, it was found that both of studies demonstrated *Cassia fistula* is helping to treat constipation among the children as shown in Table 3, but there is a risk of bias according to Cochrane Collaboration’s tool ( Table 6). Moreover, both studies found were from one country (Iran). During a methodological assessment, many flaws were identified such as inadequate patient care, attempt to blind the researcher and missing data ( Figure 2). During meta-analysis, the comparison was made before and after treatment among different variables such as defaecation, fecal incontinence, retentive posturing, the severity of pain, and consistency of stool. All the variable (before and after treatment) were found to be symmetrical when plotted on a funnel plot as shown in Figure 4, Figure 6, Figure 8, Figure 10, Figure 12, and Figure 14 respectively. The overall effect for some variables is statistically insignificant (P=0.11, P=0.49, P=0.24) such as fecal incontinence, retentive posturing, and acceptance, tolerance respectively. High heterogeneity was found in two variables i.e severity of pain (90%) and consistency of stool (77%). All the forest plot of defaecation, fecal incontinence, retentive posturing, severity of pain, consistency of stool and acceptance and tolerance are represented in Figure 3, Figure 5, Figure 7, Figure 9, Figure 11 and Figure 13 respectively.

## Conclusion

After evaluation of results, it was found that *Cassia fistula* was not completely effective. It was partly effective in reducing the pain and consistency of stool during constipation. However, these results cannot be generalized among all population. A well designed, expert validated protocol is required in the future. There is a need to develop an instrument that will be free from bias. Moreover, the results cannot be generalized among all species of *Cassia* as the studies available are only for one species. There is a need to isolate identified bioactive compounds from different species of Cassia and evaluate the effect of different factors such as duration of constipation, defecation, incontinence or retentive posturing under clinical trial.

## Declarations

### Data availability

***Underlying data.*** Open Science Framework: Application of genus Cassia in the treatment of Constipation: A systematic review. https://doi.org/10.17605/OSF.IO/PKR4N ^29^

This project contains the following underlying data:

- Cassia Senna for Constipation.rm5 (study RevMan file)
- Quotation Manager.xlsx (study characteristics of citations included in this study)

### Reporting guidelines

Open Science Framework: PRISMA diagram and flowchart for the study “Application of genus Cassia in the treatment of constipation: A systematic review”. https://doi.org/10.17605/OSF.IO/PKR4N ^29^

Data are available under the terms of the Creative Commons Zero "No rights reserved" data waiver (CC0 1.0 Public domain dedication).
