# Supplementary material for: Application of genus Cassia in the treatment of Constipation: A systematic review
Source: F1000Res. 2021 Mar 8;8:256. Originally published 2019 Mar 5. [Version 2] doi: 10.12688/f1000research.17893.2 (PMC11375415; doi:10.12688/f1000research.17893.2)
Supplement: Peer review history

# Reviewer report: Reviewer response for version 2

**DOI:** 10.5256/f1000research.55132.r316436

**Published:** 2024-09-04

**Authors:** Sara Naqvi

**Affiliations:** 1 Iqra University, Karachi, Sindh, Pakistan

This manuscript is an exemplary piece of research that makes a significant contribution to the field. The authors have presented a thorough and well-structured investigation, with clear and concise writing, impeccable methodology, and rigorous analysis. The results are robust and well-supported by the data, and the conclusions are well-founded and thought-provoking. I found no errors or weaknesses in the manuscript, and I strongly recommend it for indexing in its current form. The authors are to be commended for their outstanding work, and I have no doubt that this paper will be highly cited and influential in the field.

Are the rationale for, and objectives of, the Systematic Review clearly stated?

Yes

Is the statistical analysis and its interpretation appropriate?

Yes

If this is a Living Systematic Review, is the ‘living’ method appropriate and is the search schedule clearly defined and justified? (‘Living Systematic Review’ or a variation of this term should be included in the title.)

Yes

Are sufficient details of the methods and analysis provided to allow replication by others?

Yes

Are the conclusions drawn adequately supported by the results presented in the review?

Yes

Reviewer Expertise:

Natural products, Phytochemistry, and Gut motility.

I confirm that I have read this submission and believe that I have an appropriate level of expertise to confirm that it is of an acceptable scientific standard.

# Reviewer report: Reviewer response for version 2

**DOI:** 10.5256/f1000research.55132.r82025

**Published:** 2021-05-06

**Authors:** Massimo Bellini

**Affiliations:** 1 Gastrointestinal Unit, Department of Translational Research and New Technologies in Medicine and Surgery, University of Pisa, Pisa, Italy

I've no further particular methodological concerns. My main concern is still the same: I’m really puzzled about the usefulness of a review which takes into consideration only two papers and altogether only 132 patients.

Are the rationale for, and objectives of, the Systematic Review clearly stated?

Yes

Is the statistical analysis and its interpretation appropriate?

Yes

Are sufficient details of the methods and analysis provided to allow replication by others?

Yes

Are the conclusions drawn adequately supported by the results presented in the review?

Yes

Reviewer Expertise:

Gastroenterology: Functional Digestive Disorders.

I confirm that I have read this submission and believe that I have an appropriate level of expertise to confirm that it is of an acceptable scientific standard.

# Reviewer report: Reviewer response for version 1

**DOI:** 10.5256/f1000research.19569.r50310

**Published:** 2019-06-28

**Authors:** Farhad Shokraneh

**Affiliations:** 1 University of Nottingham, Nottingham, UK

The author investigates the effect of *Cassia fistula* on three outcomes based on two included studies. It seems a valuable research that could potentially direct the pharmaceutical companies toward a new agent to treat the constipation.

**Title**

 The title should be “Cassia fistula for treatment of children and adolescents with constipation: A systematic review of randomized controlled trials”.

**Authorship**

 The systematic review requires a process that involves at least two people for screening and data extraction. I suggest the author to find another systematic reviewer to be involved in this work.

**Abstract**

 The abstract is very brief and sentences are incomplete. Abstract should be re-written following the example from similar systematic reviews and following PRISMA for Abstract.

   

**Minor edits**

1. “act an effective as a laxative” should be “act as effective as laxatives”.
2. “search data” should be “search date”.

**Methods**

1. The standard search filter for finding RCTs as reported in Cochrane Handbook should be used.
2. It is advised in Cochrane Handbook searching Embase, MEDLINE/PubMed, and CENTRAL for systematic reviews of RCTs and this review reports searching only MEDLINE/PubMed.
3. Excluding closed access papers without trying to order them from library or contacting the authors introduces full-text or open access bias.
4. The structure of the method should change and start with 1: Eligibility Criteria 2: Databases and search strategy 3: Screening process 4: Data extraction process 5: Assessment of risk of bias process 6: Data analysis plan. Currently, the first section of the methods reports info on search and eligibility criteria while there are specific subheadings for these sections.
5. The rationale behind excluding studies older than 10 years from search date is not clear.
6. The reported number of search results 2207 is not reasonable. A proper search should not find more than 100-200 results for this topic.
7. The search date has not been reported.
8. Following a published Cochrane review of RCTs in terms of structure and all the items in PRISMA Checklist for full systematic reviews is recommended.
9. Constipation is missing from search strategy and MeSH terms in the table.
10. The numbers in PRISMA flow diagram does not match the numbers reported in the text and it does not seem to be correct. Following one of the existing published systematic reviews of RCTs from Systematic Reviews journal or Cochrane Database of Systematic Reviews is recommended.
11. The quality of included studies is being assessed using Cochrane Risk of Bias tool. The role of MQ and using Atlas.ti is not clear in this review. These two are not usually used for SR of RCTs.
12. Studying the meta-analysis section in the Cochrane Handbook which is freely available online could help revising the statistical analysis.
13. Table 2 should report PICOS not SPIO.
14. The time point for the outcome is not clear.
15. The outcome measure has not been described properly.
16. The complications and adverse effects have not been reported in the review.

**Results**

1. The sample size reported in the Data Extraction table does not match the numbers in meta-analysis. For example the total for experimental and control group in Review Manager file is 109 for Esmaeilidooki 2016 and 71 for Mozaffarpur 2012 while in the table is 51 and 81 respectively.
2. The analysis for before treatment is not required. All before treatments can be deleted.
3. Funnel plots only work with about 10-20 studies. All funnel plots can be deleted.
4. Risk of bias based on Cochrane tool is not clear. It should be low risk, unclear risk and high risk. The current description of Yes/No does not provide enough information.
5. In meta-analysis, “one randomized always analysed” while the numbers in Table 4 are not the numbers in meta-analysis in Review Manager.
6. The control groups in two studies are different so I am not sure if combining the data from two studies in a meta-analysis is a right when there is such heterogeneity in PICOS components.
7. SD is greater than mean is some outcomes which is an indicator of skewed data or error in reporting. Contacting the trials authors might make some of the points clear.

**Discussion and Conclusion**

 Current data and status of the review may not be appropriate for discussion or making a conclusion. The current evidence is limited and more rigorous studies will be required to make any conclusion or recommendations.

 I understand the author’s passion for conducting a systematic review and so I request the author to work with an expert supervisor in the field before revising the work.

Are the rationale for, and objectives of, the Systematic Review clearly stated?

Partly

Is the statistical analysis and its interpretation appropriate?

No

Are sufficient details of the methods and analysis provided to allow replication by others?

Partly

Are the conclusions drawn adequately supported by the results presented in the review?

No

Reviewer Expertise:

Evidence synthesis, Systematic review, Overview, Scoping review, Rapid review, Randomized controlled trials, Open data, Open access, Open source, Medical journalism, Peer-review, Open source, Outcomes, Interventions, Cochrane

I confirm that I have read this submission and believe that I have an appropriate level of expertise to confirm that it is of an acceptable scientific standard, however I have significant reservations, as outlined above.

# Response

**Published:** 2021-03-02

**Authors:** Muhammad Shahzad Aslam

**Affiliations:** Xiamen University Malaysia, Malaysia

WHO defines ' **Adolescents**' as individuals in the 10-19 years. So title cannot changed

# Reviewer report: Reviewer response for version 1

**DOI:** 10.5256/f1000research.19569.r47237

**Published:** 2019-05-23

**Authors:** Massimo Bellini

**Affiliations:** 1 Gastrointestinal Unit, Department of Translational Research and New Technologies in Medicine and Surgery, University of Pisa, Pisa, Italy

I’m really puzzled about the usefulness of a review which takes into consideration only two papers and altogether only 132 patients. Constipation, as the author himself states, is a very frequent condition, so the conclusions are inevitably flawed by this bias. The author should address this issue in the conclusions

 Hence, **I have significant reservations.**

 Moreover, here enclosed you'll find a copy of the paper. The authors can find some suggestions, as tracked changes and comments to the original manuscript, which could improve the quality of the manuscript.

Are the rationale for, and objectives of, the Systematic Review clearly stated?

Yes

Is the statistical analysis and its interpretation appropriate?

Yes

Are sufficient details of the methods and analysis provided to allow replication by others?

Yes

Are the conclusions drawn adequately supported by the results presented in the review?

Yes

Reviewer Expertise:

Gastroenterology: Functional Digestive Disorders.

I confirm that I have read this submission and believe that I have an appropriate level of expertise to confirm that it is of an acceptable scientific standard, however I have significant reservations, as outlined above.

# Response

**Published:** 2019-05-23

**Authors:** Muhammad Shahzad Aslam

**Affiliations:** Xiamen University Malaysia, Malaysia

Dear,

I have read the report. The selected studies were according to the criteria given inside the paper. So, there were only two selected studies for the systematic review. The comments you have mentioned are appreciable. Thanks

# Response

**Published:** 2021-03-02

**Authors:** Muhammad Shahzad Aslam

**Affiliations:** Xiamen University Malaysia, Malaysia

Please see inclusion criteria (Only articles involving children aged between 2–15 were included. Only articles involving children aged between 2–15 were included.)
